# Reduction-melting extraction of trace elements from hazardous waste glass from an old glasswork’s dump in the southeastern part of Sweden

**DOI:** 10.1007/s11356-017-0243-4

**Published:** 2017-09-24

**Authors:** Yahya Jani, William Hogland

**Affiliations:** Department of Biology and Environmental Science, Faculty of Health and Life Sciences, Linnaeus University (LNU), Landgången 3, SE-392 31 Kalmar, Sweden

**Keywords:** Crystal glass, Extraction of trace elements, Glass melting, Old glassworks dumps, Hazardous glass waste, Reduction-melting method

## Abstract

At the southeastern part of Sweden, old art and crystal waste glass has been identified as a hazardous waste due to high weight concentrations of Pb (32.398%), Cd (0.085%), and As (1.976%). The reduction-melting technique was used to investigate the extraction of these trace elements from powder waste glass of particle size < 1 mm. Following a factorial design technique, the experimental results of the reduction-melting method showed that 99.9% of Pb, 100% of Cd, and 99% of As could be extracted. For a batch of 10 g powder waste glass, the found experimental and theoretical optimum operating conditions were 1100 °C of melting temperature, 5 g of Na_2_CO_3_, 2 g of carbon, and 120 min of melting time. The reduction-melting method displayed promising results which might help in recycling the extracted trace elements and glass compared to the current used solution of landfilling as hazardous wastes.

## Introduction

In Sweden, more than 24,500 contaminated sites have been identified by the SEPA in 2016 (Swedish Environmental Protection Agency) (SEPA 2016). One thousand sites from these were classified with the highest contamination risk (class 1) for the health and environment; the Swedish criteria are based on four risk classes with the highest as class 1 and the lowest class 4 (SEPA 2016). Among these, there were 34 old glassworks sites located in the top of the list (Kalmar County Administrative Board 2016). These glassworks are located in Småland region in the southeastern part of Sweden between Kalmar and Kronoberg counties where the famous “Kingdom of Crystal glass” is located. At these glassworks, extensive amounts of trace elements were used to produce different colors and types of glasses but mostly crystal and art glass with high weight contents of Pb (more than 32%), As, Cd, Sb, Cu, Zn, and others (Hynes et al. 2004). According to Kalmar and Kronoberg county estimations, the total amount of waste glass at 25 of these glassworks was more than 500,000 t and with high contents of Pb (3100 t), As (420 t), and Cd (30 t) (Höglund et al. 2007), while the estimated total remediation cost of these 25 sites was more than 100 million € based on the old excavating and landfilling method (Höglund et al. 2007).

The contamination of former and current glassworks with different trace elements has been also reported in other parts of the world (Pant and Singh 2014) such as the glassworks at Murano island in Italy (Rossini et al. 2010) and the glassworks sites at Firozabad town in the north central India (Varun et al. 2012). The reason behind the high concentrations of trace elements in waste glass was the uncontrolled consumption of these elements as raw material in the production process which leads to the contamination of most of these glassworks and their surroundings (Varun et al. 2012; Pant and Singh 2014). Most importantly, the harmful healthy effects of these trace elements on the workers at glassworks and the people who are living around have been identified by different researchers (Apostoli et al. 1998; Pirastu et al. 1998; Rousseau et al. 2007; Sripaiboonkij et al. 2009; Brahmapurkar et al. 2013). These studies showed an increase in the frequency of lung, colon, larynx, brain, pharynx, stomach, and prostate cancers among the workers inside the glassworks and the people living close to these sites compared to the normal frequency in each country.

Unfortunately, there is a lack of information in literature about the extraction of trace elements from art and crystal waste glasses and the only proposed solution is the safe disposal in special landfills (Jani and Hogland 2014). However, landfilling waste glass means losing valuable resources that can be recycled back to the circular economy if methods of extracting trace elements from this waste can be found or developed. In the recent few years, different methods like mechano-chemical extraction (Zhang et al. 2013), enhanced leaching method (Barbieri et al. 2014; Bursi et al. 2015), volatilization process (Grause et al. 2014), vacuum metallurgical methods (Chen et al. 2009), and reduction-melting technique (Okada and Yonezawa 2013; Okada 2015; Mingfei et al. 2016) have been developed to extract Pb and other trace elements from CRT (cathode ray tube) glasses. Among these, the reduction-melting technique showed promising results of high recovery of Pb from CRT glasses with up to 99% (Mingfei et al. 2016).

The goal of the present study is to investigate the performance of the reduction-melting method to extract trace elements from waste art and crystal glasses dumped at an old glasswork site at the southeastern part of Sweden, the Pukeberg. Reducing the trace element content could be avoiding landfilling and also protecting human health and the environment. In addition, the reduction-melting process parameters were also studied statistically and experimentally to specify the optimum values that can give the highest percentage of recovery.

## Material and methods

### Site description

**Fig. 1:**
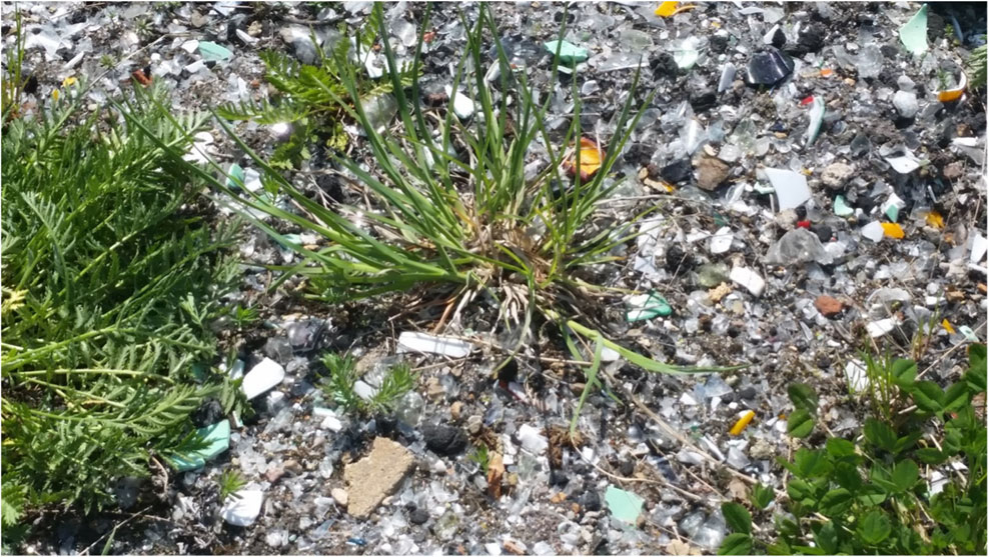
Pukeberg glasswork dumpsite and the waste glass on the surface

Pukeberg glasswork (56° 43′ N, 15° 55′ E, Sweden) was built up and started production in 1871 in Nybro municipality in the southeastern part of Sweden. The glasswork is located about 1.5 km southeast of Nybro’s municipality center, and the old glasswork’s dump (where the sampling was done) is located to the south from the glasswork buildings. The main dump covers an area of approximately 15,000 m^2^ with a maximum depth of about 2.5 m (Elert and Höglund 2012), and it contains glass wastes of different colors and ages, chemical wastes containing trace elements, and other kinds of wastes from glass production process. Waste glass can be found on the ground surface, as shown in Fig. 1.

### Sampling

The sampling points were chosen inside the Pukeberg main old dump (south of the glassworks buildings) where glass and other wastes from the daily production process were discarded between 1871 and 1950 (Höglund et al. 2007). Ten kilograms of waste glass was collected from five different points inside the main old dump and from different levels up to 50 cm. The waste glass was washed with water few times, dried at room temperature (25 °C), and then stored in closed plastic bags during conducting and performing experiments and analyses.

### Experimental setup

The waste glass was sorted and the colorless crystal glass was chosen to avoid the variation in the chemical composition of the different colors glass (Jani and Hogland 2014). Waste glass was then crushed and sieved to a particle size < 1 mm to produce a powder waste glass (PWG). This PWG was dried at 105 °C for 24 h and was used during all the reduction-melting experiments.

Ten grams of PWG was well mixed with specific weights (0–10 g) of reagent grades Na_2_CO_3_ (Sigma-Aldrich, USA) and (0–3 g) activated carbon (Sigma-Aldrich, USA). This mixture was then fed into a 50-ml high alumina crucible (Sigma-Aldrich, USA) which was placed later in an electrical furnace for a specific temperature (900–1200 °C) and heating time (30–120 min). The furnace temperature was raised according to a heating rate of 8 °C/min. Finally and after passing the melting time, the sample was left to cool to room temperature. The produced glass and aggregated trace elements were separated by crushing the crucible and the produced glass, as shown in Fig. 7.

The reduction-melting experiments were done by following a factorial design technique by changing one parameter each time from the four studied parameters: melting temperature (900–1200 °C), weight of Na_2_CO_3_ (0–10 g), weight of carbon (0–3 g), and melting time (30–120 min). However, the reduction-melting experiment was considered successful only in case when the participating trace elements could be seen with the naked eyes in the final products.

### Analyses

A semi-quantitative X-ray fluorescence analysis (XRF), Olympus DS-4000 (Innov-X system, USA), was used to find the chemical composition of the PWG and the produced glass and trace elements after each experiment.

The following equation was used to calculate the recovery percentage of the trace elements (Okada et al. 2012; Okada 2015):

1 $$ \mathrm{Trace}\  \mathrm{element}\  \mathrm{recovery}\%=\left(1-\frac{\frac{\mathrm{final}\  \mathrm{concentration}\  \mathrm{of}\  \mathrm{trace}\  \mathrm{element}}{\mathrm{final}\  \mathrm{concentration}\  \mathrm{of}\  \mathrm{silica}}}{\frac{\mathrm{initial}\  \mathrm{concentration}\  \mathrm{of}\  \mathrm{trace}\  \mathrm{element}}{\mathrm{initial}\  \mathrm{concentration}\  \mathrm{of}\  \mathrm{silica}\ }}\right)\times 100 $$

where final concentrations of trace element and silica are the concentrations after each reduction-melting experiment, while the initial concentration means the concentration in the PWG. The purpose behind using this equation is to eliminate the dilution effects of the Na_2_CO_3_ on the trace elements.

#### Leaching test

The reduction-melting glass residuals at the optimum conditions were crushed and sieved to particle sizes of less than 2 mm. The leachate was prepared by mixing 5 g of glass with 50 ml distilled water in one stage shaking leaching test (liquid to solid ratio (L/S) of 10) for 24 h by following the Nordtest method NT ENVIR 004. Then, the leachate was analyzed by ICP-MS to find the trace elements content according to the EN 12457-3 standard.

## Results and discussions

### Chemical composition

The chemical composition of the PWG compared to the Swedish limits (Avfall Sverige 2007) of hazardous wastes is shown in Table 1. The results showed that the PWG was identified as hazardous waste due to the high concentrations of Pb, Cd, and As. However, high concentrations of Sb, Cu, and Zn were also found but with concentrations less than that of the Swedish guidelines. For the safe recycling or even landfilling of Pukeberg waste glass as inert material, the concentrations of Pb, Cd, and As must be reduced by extracting these trace elements to accepted levels.

**Table 1:** The chemical composition of the sampled waste glass used in the present study (PWG) compared to the Swedish limits of hazardous waste (Avfall Sverige 2007)

Element (mg/kg)	Mean value of the PWG, present study	Swedish limits of hazardous wastes (Avfall Sverige 2007)
Si	438,241 (3547)	
S	132,508 (2896)	
Cl	12,202 (422)	
K	52,494 (825)	
Cr	54 (7)	1000
Ca	4294 (148)	
Mn	137 (7)	
Fe	538 (111)	
Cu	856 (69)	2500
Zn	1616 (106)	2500
As	19,761 (705)	1000
Mo	44 (5)	10,000
Cd	847 (38)	100
Sn	581 (44)	
Sb	4373 (108)	10,000
Ba	878 (30)	10,000
Pb	323,977 (8524)	2500

Note: values between brackets represent the standard deviation. And the red color means that the found value was higher than that of the Swedish limits of hazardous waste

On the other hand, the comparison between the chemical composition of PWG and CRT glass (Mear et al. 2006) is shown in Table 2. The concentrations of Pb, As, and Zn were higher in PWG compared to CRT glass. In reverse, the concentrations of Si, K, Ca, Fe, Sb, and Ba were slightly higher in CRT glass. Some trace elements like Cr and Cu appear only in PWG while others like Al appear only in CRT glass. In general, CRT and crystal glasses are known as lead glasses because the chemical composition is dominated by Pb (BREF 2009). Hence, methods used to extract trace elements from CRT glass could be also used for crystal glass due to the similarity in the chemical and physical properties (Vogel 1994; Shelby 2005; BREF 2009).

**Table 2:** PWG glass chemical composition compared to CRT glass based on weight percentage

Element (wt%)	Mean value of the PWG, present study	Mean value of CRT glass (Mear et al. 2006)
Si	43.82	49.77
S	13.25	
Cl	1.22	
K	5.25	7.31
Cr	0.01	
Ca	0.43	3.56
Mn	0.01	
Fe	0.05	0.1
Cu	0.09	
Zn	0.16	0.03
As	1.98	0.01
Mo	0.01	
Cd	0.08	
Sn	0.06	
Sb	0.44	0.57
Ba	0.09	0.36
Pb	32.40	22.90
Al		3.28
Others	0.65	12.11

### Effect of the reduction-melting parameters on the trace elements recovery

According to different studies (Okada and Yonezawa 2013; Okada 2015; Mingfei et al. 2016), the reduction-melting method depends on four parameters that plays vital role in the extraction of trace elements from CRT glasses. These parameters are the melting temperature, Na_2_CO_3_ weight, carbon dosage, and melting time. However, to investigate the effect of these parameters on the percentage of recovery of Pb, Cd, and As and to find the optimum values with the highest recovery, these parameters were studied experimentally and theoretically as follows.

#### Melting temperature

**Fig. 2:**
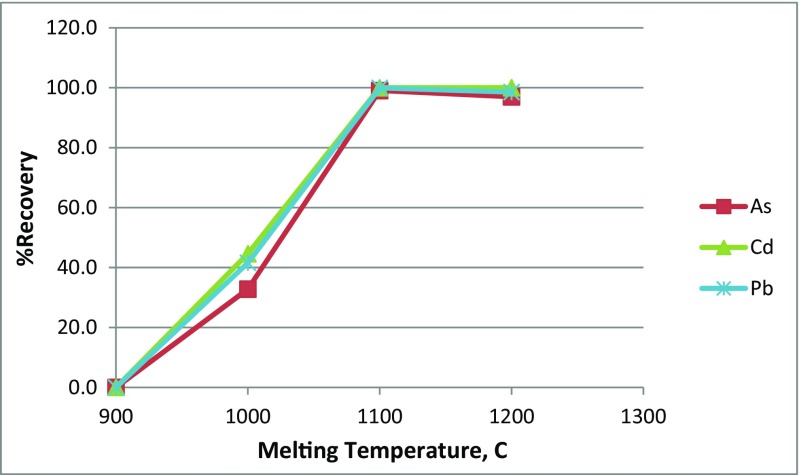
The effect of melting temperature on the percentage recovery of Pb, Cd, and As from the studied PWG

Due to the homogenous chemical structure of glass and the strong bonded of silicon oxide to the trace elements oxides, high temperature, higher than 900 °C, was needed for the melting of glass and releasing the trace elements from the glass network structure (Singh et al. 2016). However, this was enhanced by the addition of Na_2_CO_3_ and carbon, as will be discussed later. Figure 2 shows the effect of melting temperature on the percentage of recovery of Pb, Cd, and As. The results showed that increasing the temperature increased the recovery of the three trace elements linearly up to 1100 °C. According to Stokes’ law, the separation efficiency of the metallic trace elements depends on the sedimentation velocity which in turn depends on the following:

2 $$ V=\frac{\left({\rho}_{\mathrm{s}}-\rho \right)g{d}_{\mathrm{s}}^2}{18\mu } $$

where *V* is the sedimentation velocity of the metallic trace elements’ particle, *ρ*_s_ and *ρ* are the densities of metallic trace element and glass, respectively, *g* is the gravitational acceleration, *d*_s_ is the particle diameter of the metallic trace elements, and *μ* is the glass viscosity. The density difference (*ρ*_s_ − *ρ*) between the metallic trace elements Pb (11.3 g cm^−3^), Cd (8.7 g cm^−3^), and As (5.7 g cm^−3^) and glass (3 g cm^−3^) (Green and Perry 2007) is acting as the driving force to accelerate the sedimentation velocity. Moreover, the sedimentation velocity increased by decreasing glass viscosity due to the high temperature and the addition of Na_2_CO_3_ that modify the network structure of glass by breaking the SiO_2_ and releasing the mobile SiO_4_ (Vogel 1994).

At 900 °C, no aggregates of trace elements were noticed with the naked eyes in the final products of the melting experiments and the percentage of recovery was considered zero. In addition, the recovery results at 1100 and 1200 °C were almost the same for the three trace elements which means that studying the melting temperature further at temperatures higher than 1200 °C was not relevant. The highest percentage of recovery of Pb (99.9%), Cd (100%), and As (99%) was found at 1100 °C; this result was in agreement with that of Li et al. (2012) and Okada et al. (2012) for the recovery of Pb from CRT glass.

#### Addition of Na_2_CO_3_

**Fig. 3:**
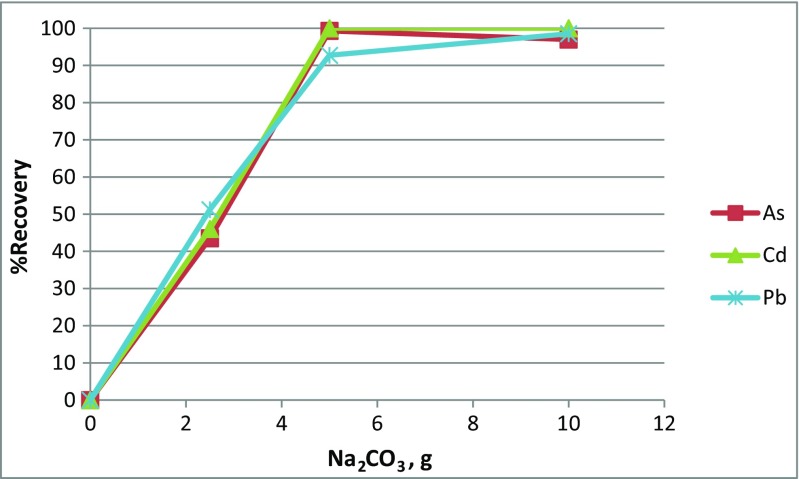
The effect of Na_2_CO_3_ weight on the percentage of recovery of Pb, Cd, and As from the studied PWG

The effect of the addition of Na_2_CO_3_ (flux) on the recovery percentage of Pb, Cd, and As is shown in Fig. 3. The percentage of recovery of the three trace elements increased with increasing the Na_2_CO_3_ weight up to 5 g for each 10 g of PWG. Then, increasing the Na_2_CO_3_ further did not affect the percentage of recovery. At temperatures higher than 600 °C, the Na_2_CO_3_ will be dissociated to CO_2_ and Na_2_O, shown in the chemical reaction 1, which acts as a glass network modifier (Mingfei et al. 2016):

3 $$ {Na}_2{CO}_3={Na}_2O+{CO}_2 $$

As can be shown in the chemical reactions 2–4, the Na_2_O will replace the PbO in the PbO-SiO_2_ structure due to the higher strength of the Na–O bond compared to that of the Pb–O resulting in decreasing the molten glass viscosity and promoting the trace elements precipitation (Li et al. 2012). Additionally, the produced CO_2_ (with the help of the added carbon) will reduce the PbO to produce metallic lead at temperature higher than 900 °C and the reduction rate increased quickly with increasing the temperature (Xing and Zhang 2011).

4 $$ {Na}_2\mathrm{O}+ PbO-\mathrm{nSi}{\mathrm{O}}_2={Na}_2\mathrm{O}-n\mathrm{Si}{\mathrm{O}}_2+ PbO $$

5 $$ C+C{\mathrm{O}}_2=2 CO $$

6 $$ PbO+ CO= Pb+{CO}_2 $$

Moreover, the same mechanism will also enhance the reduction of Cd and As oxides (Li et al. 2012). The same results were also found by Li et al. (2012) and Okada and Yonezawa (2013) but for the CRT glass.

#### Carbon addition

**Fig. 4:**
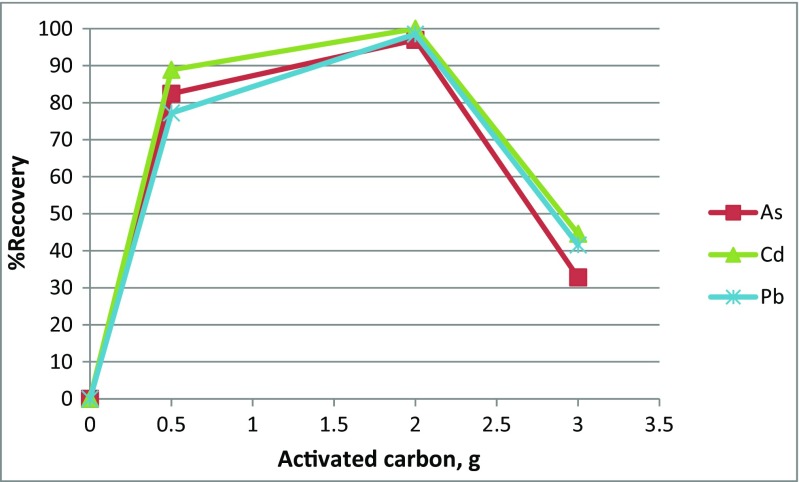
The effects of carbon dosage on the recovery rate of Pb, Cd, and As from PWG

Carbon is considered as one of the best and most used reducing agents in metallurgy processes like in the production of steal (Mingfei et al. 2016). The carbon itself and the CO has the ability to reduce trace elements oxides to metallic form like in the case of Pb, Cd, and As according to the chemical reactions (4) and (5):

7 $$ 2 MO+2 CO=2M+2{CO}_2 $$

where M means trace element and MO trace element oxide. As shown in Fig. 4, the percentage of trace elements recovery increased with increasing the carbon weight till reaching the maximum recovery at 2 g of carbon for each 10 g of PWG then the recovery rate decreased with increasing the weight of carbon further. This might be due to the incomplete burning of carbon as was noticed in the final product of the 3 g experiment. The same result was also found by Okada et al. (2012) for CRT glass.

#### Melting time

**Fig. 5:**
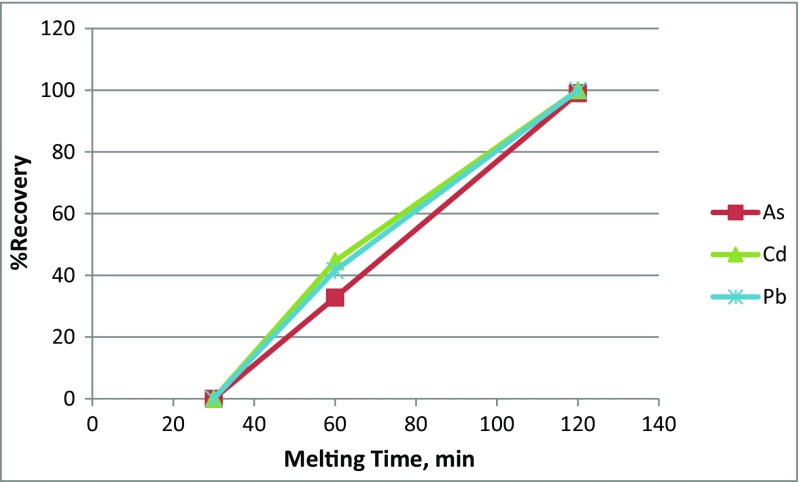
The effect of melting time of the percentage of recovery of Pb, Cd, and As from PWG

Figure 5 shows the effect of melting time on the percentage of recovery of Pb, Cd, and As from PWG. The results showed that the recovery increased linearly with increasing the melting time reaching the maximum recovery of Pb (99.9%), Cd (100%), and As (99%) at 120 min. Okada and Yonezawa (2013) showed that the percentage of recovery was controlled by the reduction rate of trace elements oxides; it means that when the time increases, further the percentage of recovery decreased due to the reduction in the concentrations of the trace elements oxides (Okada and Yonezawa 2013). In addition, the size of the precipitating trace elements particle (*d* ^2^_s_) increased with increasing the melting time as was noticed between the particles sizes at 60- and 120-min experiments. This could also enhance the separation efficiency of the trace elements due to the acceleration in the sedimentation velocity that was explained earlier by the Stokes’ law.

### Statistical analyses

**Fig. 6:**
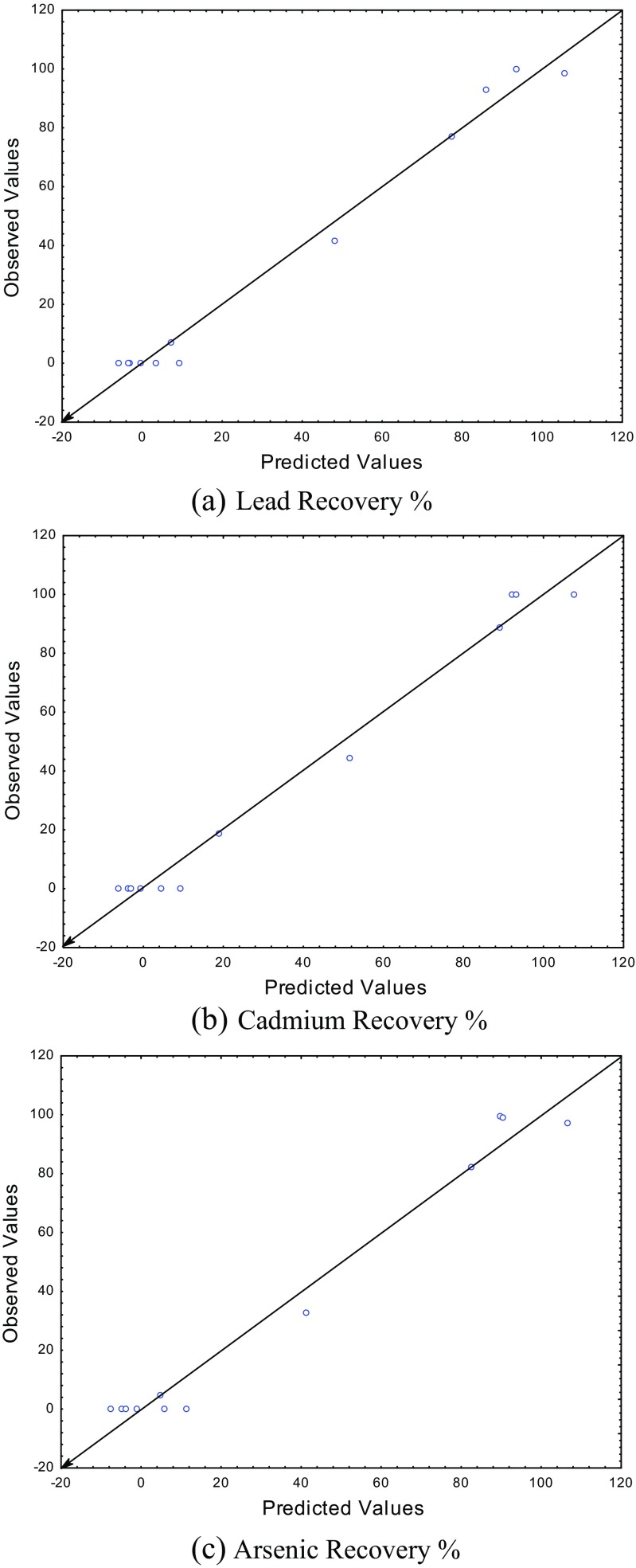
The observed (experimental) versus the predicted (theoretical calculation) values of the percentage recovery of **a** lead, **b** cadmium, and **c** arsenic

**Fig. 7:**
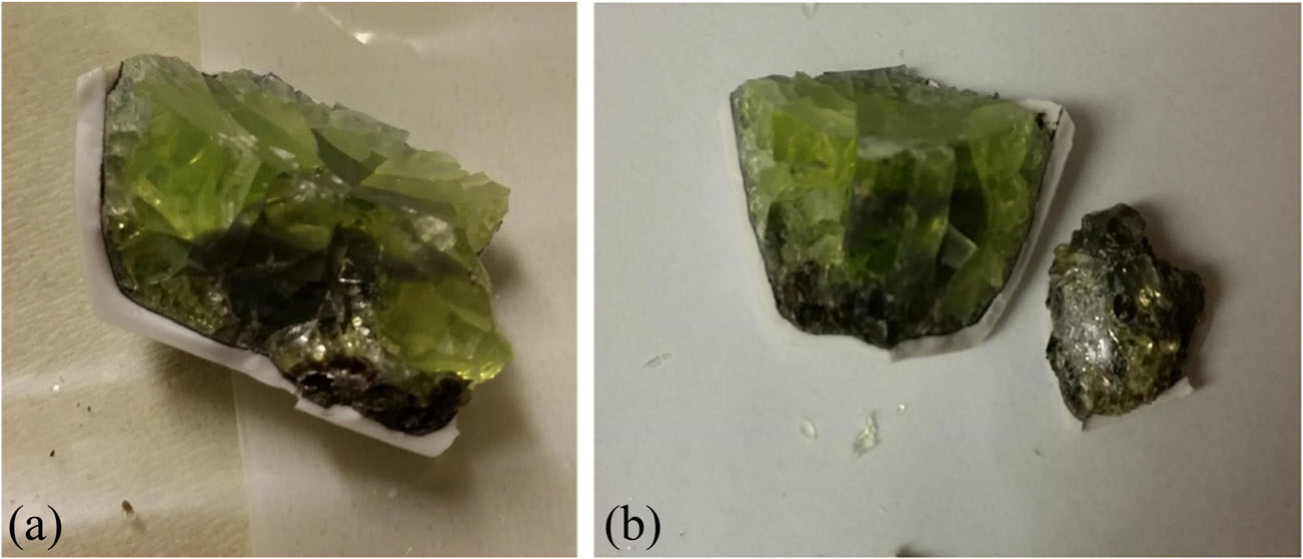
The final product of the reduction-melting experiment at the optimum values of the studied parameters 1100 °C of temperature, 2 g of carbon, 5 g of Na_2_CO_3_, and 120 min of melting time. **a** Before crushing and separating. **b** After crushing

A theoretical polynomial model was used to find the correlation between the reduction-melting process parameters and their effects on the percentage recovery of the studied trace elements Pb, Cd, and As. However, the same model was used to find the theoretical optimum values of the studied parameters and compared them with the experimental results by finding the correlation coefficient for each trace element. The used polynomial model was as follows:

$$ Y={a}_0+{a}_1{X}_1+{a}_2{X}_2+{a}_3{X}_3+{a}_4{X}_4+{a}_5{X}_1^2+{a}_6{X}_2^2+{a}_7{X}_3^2+{a}_8{X}_4^2+{a}_9{X}_1{X}_2{X}_3{X}_4 $$

where *Y* is the percentage of recovery, *X*_1_ is the melting temperature in degree Celsius, *X*_2_ is the carbon weight in grams, *X*_3_ is the Na_2_CO_3_ weight in grams, *X*_4_ is the melting time in minutes, and the *a*_(0–9)_ is the model constants. To find the model constants and the correlation coefficients for the recovery percentage of each trace element, a STATISTICA program version 6 was used. As shown in Table 3, high correlation coefficients were found for the recovery of the three trace elements according to the proposed model equation with 99.2, 99.1, and 98.6% for Pb, Cd, and As, respectively. This means that these parameters are highly interconnected and they are playing vital role in the recovery of the trace elements from the studied waste glass. In addition, the statistical results (using least square method) showed that the experimental and theoretical optimum values of the four parameters were the same with melting temperature of 1100 °C, 2 g of carbon, 5 g of Na_2_CO_3_, and for 120 min of melting time, as shown in Fig. 6. These optimum values gave the highest percentage of recovery of Pb (99.9%), Cd (100%), and As (99%), with the final result shown in Fig. 7.

**Table 3:** The polynomial model equations of the percentage of recovery of Pb, Cd, and As from the studied PWG with the correlation confidents

Trace element	Model equation	Correlation coefficient (%)
Pb	$Y=-1404.25+1.77{X}_1+85.68{X}_2+24.23{X}_3+4.99{X}_4+0.0007{X}_1^2-20.22{X}_2^2-1.14{X}_3^2-0.03{X}_4^2-0.00001{X}_1{X}_2{X}_3{X}_4$	99.2
Cd	$Y=-1348.28+1.74{X}_1+62.2{X}_2+25.54{X}_3+4.4{X}_4+0.0006{X}_1^2-15.51{X}_2^2-1.35{X}_3^2-0.02{X}_4^2-0.00007{X}_1{X}_2{X}_3{X}_4$	99.1
As	$Y=-1205.09+1.42{X}_1+73.07{X}_2+24.02{X}_3+4.96{X}_4+0.0005{X}_1^2-18.35{X}_2^2-1.23{X}_3^2-0.03{X}_4^2-0.00008{X}_1{X}_2{X}_3{X}_4$	98.6

### Leachate trace element content

The leachate trace element content of the residual glass at the optimum operating conditions compared to the European landfill guidelines (European Union 2003) for inert and hazardous waste is shown in Table 4. The results showed that the reduction-melting method could reduce the concentrations of all the trace elements, but still, the concentrations were higher than the European guidelines of landfilling as inert and the As and Sb concentrations were double and triple higher than the European guidelines of landfilling as hazardous wastes. This could be due to the mobilization of the trace elements in the glass network structure after the reduction-melting experiments (Okada et al. 2012). To stabilize these elements in the glass network structure and reducing the leaching effects, the residual glass was re-melted at 1200 °C for 1 h and the leaching test was repeated after crushing the glass residual to particle sizes less than 2 mm. The results, shown in Table 4, indicated that the re-melting could reduce the leaching of all the trace elements to levels less than that of the European guidelines of landfilling as inert waste (European Union 2003). This could be explained by the oxidation of the metallic trace elements which leads to the re-connection of these oxides back in the SiO_2_ glass network structure as was also noticed by Okada et al. (2012). However, more experimental work is needed in future to explore the re-melting stability performance.

**Table 4:** Leachate trace elements content of residual glass at the reduction-melting optimum conditions compared to the European Union guidelines (European Union 2003) of landfilling as inert and hazardous wastes at liquid to solid ratio of 10

Element (mg/kg)	Mean value of residual glass before re-melting, present study	Mean value of residual glass after re-melting, present study	Limits of landfilling as inert waste (European Union 2003)	Limits of landfilling as hazardous waste (European Union 2003)
Cr	0	0	10	70
Mn	0	0	–	–
Cu	0	0	50	100
Zn	0.3	0.01	50	200
As	45.8	0.07	2	25
Cd	0	0	1	5
Sb	15.6	0.06	0.7	5
Ba	0.2	0	100	300
Pb	49	0.01	10	50

### Trace element mass balance

At high operating temperature (900–1200 °C), there is a probability for the evaporation of some trace elements. Therefore, the mass balance of the trace element content of the PWG, the glass residual, and the separated trace elements (shown in Table 5) was analyzed. The found differences were less than 2% and within the XRF experimental analyses errors (Okada 2015).

**Table 5:** The XRF analysis of the extracted trace elements at the optimum reduction-melting operating conditions displayed in weight percentages

Element (wt%)	Extracted trace elements, the present study
Cr	0.02
Mn	0.03
Cu	2.19
Zn	0.14
As	4.83
Cd	0.12
Sb	0.94
Ba	0.15
Pb	91.58

### Recycling potential

Reducing the trace element content of the Swedish art and crystal waste glasses can enhance the recycling of both the extracted trace elements and the remaining glass. Recycling these waste materials, which were considered as hazardous due to the high weight concentrations of Pb, Cd, and As, is important for protecting human health and the environmental and for the sustainability of the Earth natural resources that have been used extensively in glass industry (Jani and Hogland 2014). Furthermore, the European circular economy goals emphasized on considering all the waste materials, like wastes glass, as valuable resources that must be recovered and recycled back. The reduction-melting process showed promising results in minimizing the hazardous trace elements in the studied waste glass which might help in using these glasses as raw material in the production of new crystal and art glass, in cement and construction industry (Jani and Hogland 2014), glass ceramics (Kritikaki et al. 2016), or even in the production of foam glass (Guo et al. 2010).

On the other hand, the extracted trace elements (Pb, Cd, and As) can be also recycled like in battery industry especially the lead, due to the high weight content (32.398%). Methods like electrochemical, supercritical, and vacuum metallurgical technologies have been used for the purification of trace elements like lead (Zhang and Xu 2016). However, recycling the extracted trace elements and glass must be studied carefully in future in order to specify the safe use and the requirements of the raw materials of the target industries.

## Conclusions

The Kingdom of Crystal glass in the southeastern part of Sweden is well known by the production of the famous art and crystal glass. The uncontrolled consumption of trace elements as raw materials in the production process leads to hazardous contamination of these glasses with high weight concentrations of Pb (32.398%), Cd (0.085%), and As (1.976%). The reduction-melting method was used to extract these trace elements from sampled waste glass from the dumpsite at Pukeberg glasswork. The experimental results of the followed factorial design of experiments showed that the percentage of recovery of the trace elements increased with increasing melting temperature, Na_2_CO_3_, carbon, and melting time. In addition, the experimental and theoretical results displayed that these four parameters were highly interconnected and played vital role in the reduction-melting process. The optimum melting temperature (1100 °C), Na_2_CO_3_ weight (5 g), carbon (2 g), and melting time (120 min) were found experimentally and theoretically with high percentage of recovery of Pb (99.9%), Cd (100%), and 99% of As. The findings of the present study may help in recycling the extracted glass and trace elements instead of the proposed governmental plan by excavating and landfilling as hazardous wastes in special landfills.
